# Associations between model-predicted rivaroxaban exposure and patient characteristics and efficacy and safety outcomes in patients with non-valvular atrial fibrillation

**DOI:** 10.1007/s11239-020-02077-9

**Published:** 2020-04-23

**Authors:** Liping Zhang, Xiaoyu Yan, Keith A. A. Fox, Stefan Willmann, Partha Nandy, Scott D. Berkowitz, Anne Hermanowski-Vosatka, Jeffrey I. Weitz, Alexander Solms, Stephan Schmidt, Manesh Patel, Gary Peters

**Affiliations:** 1 Janssen Research & Development, LLC, Raritan, NJ USA; 2 Centre for Cardiovascular Science, The University of Edinburgh, Edinburgh, UK; 3 Clinical Pharmacometrics, Bayer AG, Wuppertal, Germany; 4 Bayer U.S., LLC, Research & Development, Pharmaceuticals, Whippany, NJ USA; 5 Thrombosis & Atherosclerosis Research Institute, McMaster University, Hamilton, ON Canada; 6 Clinical Pharmacometrics, Bayer AG, Berlin, Germany; 7 Center for Pharmacometrics and Systems Pharmacology, Department of Pharmaceutics, College of Pharmacy, University of Florida, Orlando, FL USA; 8 Duke Clinical Research Institute, Durham, NC USA; 9 Clinical Pharmacology and Pharmacometrics, Janssen Research & Development, LLC, 920 Route 202, Raritan, NJ 08869 USA

**Keywords:** Atrial fibrillation, Drug monitoring, Pharmacokinetics, Randomized controlled trial, Rivaroxaban

## Abstract

Rivaroxaban exposure and patient characteristics may affect the rivaroxaban benefit–risk balance. This study aimed to quantify associations between model-predicted rivaroxaban exposure and patient characteristics and efficacy and safety outcomes in patients with non-valvular atrial fibrillation (NVAF), using data from the phase 3 ROCKET AF trial (NCT00403767). In ROCKET AF, 14,264 patients with NVAF were randomized to rivaroxaban (20 mg once daily [OD], or 15 mg OD if creatinine clearance was 30–49 mL/min) or dose-adjusted warfarin (median follow-up: 707 days); rivaroxaban plasma concentration was measured in a subset of 161 patients. In this post hoc exposure–response analysis, a multivariate Cox model was used to correlate individual predicted rivaroxaban exposures and patient characteristics with time-to-event efficacy and safety outcomes in 7061 and 7111 patients, respectively. There was no significant association between model-predicted rivaroxaban trough plasma concentration (C_trough_) and efficacy outcomes. Creatinine clearance and history of stroke were significantly associated with efficacy outcomes. C_trough_ was significantly associated with the composite of major or non-major clinically relevant (NMCR) bleeding (hazard ratio [95th percentile vs. median]: 1.26 [95% confidence interval 1.13–1.40]) but not with major bleeding alone. The exposure–response relationship for major or NMCR bleeding was shallow with no clear threshold for an acceleration in risk. History of gastrointestinal bleeding had a greater influence on safety outcomes than C_trough_. These results support fixed rivaroxaban 15 mg and 20 mg OD dosages in NVAF. Therapeutic drug monitoring is unlikely to offer clinical benefits in this indication beyond evaluation of patient characteristics.

**Electronic supplementary material:**

The online version of this article (10.1007/s11239-020-02077-9) contains supplementary material, which is available to authorized users.

## Highlights

- In these post hoc exposure–response analyses conducted for the rivaroxaban arm of ROCKET AF, there was no significant relationship between predicted rivaroxaban exposure and efficacy outcomes.
- There was a significant relationship between rivaroxaban exposure and the incidence of major or non-major clinically relevant (NMCR) bleeding, but the association between exposure and the risk of major bleeding was not statistically significant.
- The increase in the risk of NMCR bleeding with increasing exposures was gradual and the exposure–response relationship shallow, with no clear threshold for acceleration of bleeding risk.
- Patient characteristics had a greater impact on efficacy outcomes and the risk of major bleeding, and a similar or greater influence on the risk of major or NMCR bleeding, compared with rivaroxaban exposure.
- These findings suggest monitoring rivaroxaban levels is unlikely to offer benefits over evaluating patient factors.

## Introduction

Rivaroxaban, an oral direct factor Xa inhibitor, is approved for the prevention of stroke and systemic embolism (SE) in adults with non-valvular atrial fibrillation (NVAF) with one or more risk factors (e.g., prior stroke) [1], based on the phase 3, randomized, controlled trial ROCKET AF (NCT00403767) [2]. In ROCKET AF, rivaroxaban (20 mg once daily [OD], or 15 mg OD if creatinine clearance [CrCl] was 30–49 mL/min) was non-inferior to dose-adjusted warfarin for the prevention of stroke or SE, and similar with respect to the risk of major bleeding or a composite of major or non-major clinically relevant (NMCR) bleeding.

Advanced age and impaired renal function are associated with increased rivaroxaban exposure [1] and are also independent risk factors for NVAF-related thromboembolism and for major bleeding events in anticoagulant-treated patients [3–6]. It has been proposed that therapeutic drug monitoring (i.e., plasma concentration-based dose adjustment) may help guide anticoagulant dosing for individual patients. This post hoc exposure–response analysis aimed to explore this possibility and to quantify the associations between predicted rivaroxaban exposures, patient characteristics and clinical outcomes in patients with NVAF using data from ROCKET AF.

## Methods

### Study design

Full details of the methodology and ethical conduct of the ROCKET AF study have been reported previously [2, 7]. Briefly, 14,264 patients with NVAF were randomized to receive rivaroxaban (20 mg OD, or 15 mg OD in patients with a CrCl of 30–49 mL/min) or dose-adjusted warfarin (median follow-up: 707 days; median duration of treatment: 590 days) (Table 1) [2, 7].

**Table 1:** Description of ROCKET AF and outcomes and event rates for the exposure–response analyses

	ROCKET AF [2]
Design	Multicenter, randomized, double-blind, double-dummy, event-driven trial conducted at 1178 participating sites in 45 countries
Population	Patients with NVAF, as documented on electrocardiography, who were at moderate-to-high risk of stroke
Total number of patients randomized	14,264
Pertinent exclusion criteria	Hemodynamically significant mitral valve stenosis, planned electrical or pharmacological cardioversion, active internal bleeding, history of, or a condition associated with, increased bleeding risk
Rivaroxaban dose and regimen	20 mg OD, or 15 mg OD in patients with a CrCl of 30–49 mL/min
Comparator dose and regimen	Adjusted-dose warfarin (target INR, 2.0–3.0)
Other treatments	Concomitant use of aspirin was allowed
Median follow-up	707 days
Median treatment duration	590 days
Total number of patients for ER analysis	7061 (efficacy) 7111 (safety)
Efficacy outcomes for ER analysis: n (%)	1. Ischemic stroke or non-CNS SE: 154 (2.2) 2. Ischemic stroke, non-CNS SE or all-cause death: 357 (5.1)
Safety outcomes for ER analysis: n (%)	1. Major bleeding^a^: 395 (5.6) 2. Major or NMCR bleeding^b^: 1457 (20.7)

*CNS* central nervous system, *CrCl*, creatinine clearance, *ER* exposure–response, *INR* international normalized ratio, *NMCR* non-major clinically relevant, *NVAF* non-valvular atrial fibrillation, *OD* once daily, *SE* systemic embolism

^a^Major bleeding was defined, in accordance with International Society on Thrombosis and Haemostasis criteria, as the following: overt bleeding associated with a decrease in hemoglobin level of ≥ 2 g/dL or leading to a transfusion of ≥ 2 units of packed red blood cells or whole blood; bleeding in a critical site; or bleeding contributing to death [24]

^b^NMCR bleeding was defined as overt bleeding that did not meet the criteria for major bleeding but that was associated with medical intervention, unscheduled contact with a physician, interruption or discontinuation of study drug, or discomfort or impairment of activities of daily life [2]

The efficacy outcomes evaluated in this exposure–response analysis were a composite of ischemic stroke or non-central nervous system (non-CNS) SE, and a composite of ischemic stroke, non-CNS SE or all-cause death. Major bleeding events and the composite endpoint of major or NMCR bleeding events were evaluated as safety outcomes (Table 1).

### Patient characteristics

Patient characteristics for potential inclusion in the exposure–response evaluation were identified a priori based on a review of the literature [8–11] and experiences in ROCKET AF [2, 12, 13]. The variables were categorical in nature or grouped categorically to aid clinical interpretation.

### Rivaroxaban exposure predictions

An integrated population pharmacokinetics (popPK) model was developed as previously described [14]. The model used pooled rivaroxaban pharmacokinetic data from a subset of 161 patients for whom rivaroxaban exposure was measured in ROCKET AF, and from patients in six phase 2 trials of rivaroxaban in which a wide range of rivaroxaban doses were evaluated [14]. Individual steady-state rivaroxaban exposure metrics (including area under the plasma concentration–time curve from time 0 to 24 h [AUC_0–24_], maximum plasma concentration [C_max_] and trough plasma concentration [C_trough_]) for each patient were predicted based on individual patient characteristics (age, weight, renal function measured as rate of CrCl, and sex) and rivaroxaban dose.

Using patient characteristics alone to predict individual exposure might not appropriately reflect the variability expected. Therefore, prothrombin time (PT) measurements, collected from ROCKET AF participants at weeks 12 and 24, were used to derive rivaroxaban AUC_0–24_, C_max_ and C_trough_, based on the linear relationship between plasma concentration and PT determined using a thromboplastin reagent sensitive to the anticoagulant effects of rivaroxaban [15]. This adjustment enhanced precision in the exposure predictions and was applied to 5681 patients in ROCKET AF, including the 161 patients with available rivaroxaban exposure measurements [15].

Exposure–efficacy analyses included patients who received at least one dose of rivaroxaban, were followed for events while receiving rivaroxaban or within 2 days after discontinuation, and had available efficacy outcome data. Exposure–safety analyses included patients who received at least one dose of rivaroxaban and were followed for events while receiving rivaroxaban or within 2 days after discontinuation. Measures of exposure in these analyses were predicted based on the popPK model, patient characteristics and dose, with or without PT adjustment for over 7000 patients.

### Regression analyses

Relationships between rivaroxaban exposure metrics, patient characteristics and the efficacy and safety outcomes were assessed using Cox proportional regression analysis, as described in the supplemental material. The hazard ratios (HRs) generated for the variables using the final models for each outcome were displayed in forest plots. The reference category was the category most commonly observed for the variable, except for geographic region for which Western Europe was set as the reference. The final models were used to simulate the probability of efficacy or safety events at 1 year versus predicted exposure in a typical patient population (i.e., with individual patient characteristics set to reference values).

## Results

### Patient characteristics

Supplemental Table 1 shows the characteristics of patients selected for evaluation in the efficacy (n = 7061) and safety (n = 7111) populations. Approximately 38% of patients were > 75 years of age, 40% were female, 81% had persistent atrial fibrillation (AF) and 43% had a CHADS_2_ score of 3. Baseline antiplatelet and non-steroidal anti-inflammatory drug (NSAID) use and prior vitamin K antagonist use were reported in 40%, 4% and 62% of patients, respectively. Histories of stroke, transient ischemic attack and SE were present in 34%, 22% and 4% of patients, respectively. Baseline CrCl was < 50 mL/min in 21% of patients.

### Rivaroxaban exposure predictions and event rates

Predicted C_trough_ showed larger between-patient variability than predicted AUC_0–24_ or C_max_ (Supplemental Table 2). The exposure predictions were all highly correlated (> 0.85) within a given individual. The observed event rates for efficacy and safety outcomes are summarized in Table 1.

C_trough_ was the exposure metric most strongly associated with the likelihood of both efficacy and safety events, as evident from the lowest Akaike information criterion (AIC) value, and was selected for investigation for both analyses, as described in the supplemental material.

### Regression analyses

The results of the final exposure–response models are shown in Table 2 and Supplemental Table 3.

**Table 2:** Results of the final exposure–response models

Variables	Efficacy		Safety	
	Ischemic stroke and non-CNS SE	Ischemic stroke, non-CNS SE and all-cause death	Major bleeding	Major/NMCR bleeding
Age^a^	n.s	n.s	X	X
CrCl^a^	X	X	n.s	n.s
Best exposure	n.s	n.s	n.s	C_trough_
Other significant covariate	History of stroke	History of HF History of MI Geographic region History of stroke	Aspirin use^b^ History of GI bleeding Low hemogloblin^b^ NSAID use^b^ Geographic region	Antiplatelet use^b^ History of GI bleeding Low hemoglobin^b^ Geographic region History of vascular disease

*CNS* central nervous system, *CrCl* creatinine clearance, *C*_*trough*_ trough plasma concentration, *GI* gastrointestinal, *HF* heart failure, *MI* myocardial infarction, *NMCR* non-major clinically relevant, *n.s*. not significant, *NSAID* non-steroidal anti-inflammatory drug, *SE* systemic embolism

^a^Forced input variables

^b^At baseline

X denotes statistically significant exposure–response relationship (p ≤ 0.01)

#### Exposure–efficacy analysis

**Fig. 1:**
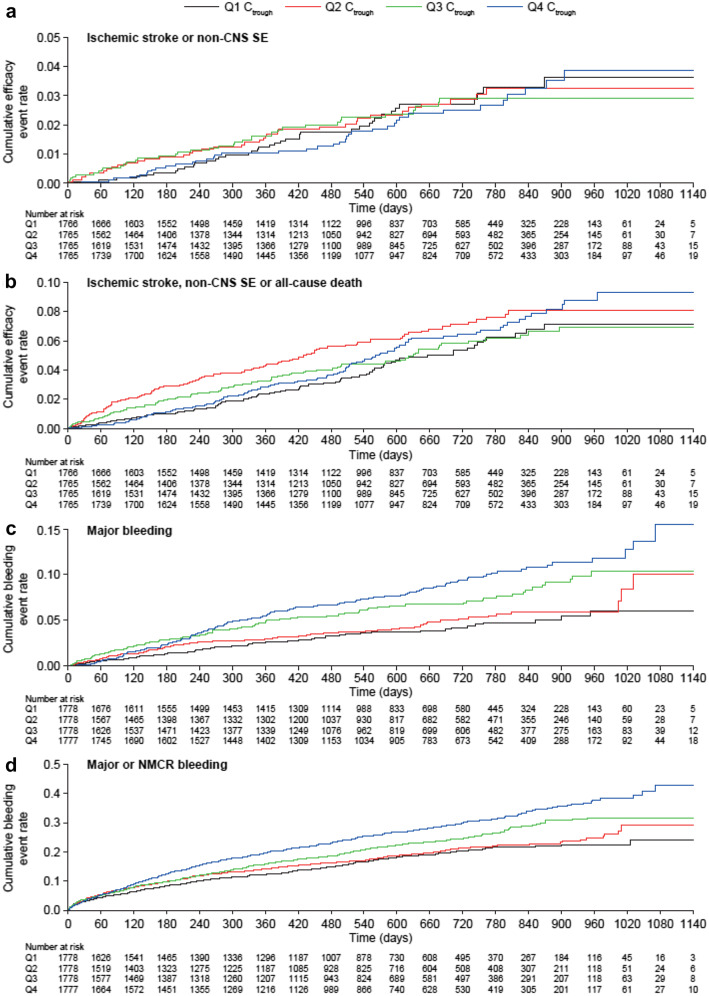
Kaplan–Meier plots of the cumulative event rates for the following outcomes versus predicted steady-state C_trough_: composite efficacy outcomes of (**a**) ischemic stroke or non-CNS SE and (**b**) ischemic stroke, non-CNS SE or all-cause death; and the safety outcomes of (**c**) major bleeding and (**d**) major or NMCR bleeding. *CNS* central nervous system, *C*_*trough*_ trough plasma concentration, *NMCR* non-major clinically relevant, *Q* quartile, *SE* systemic embolism

There was no apparent trend between C_trough_ quartiles and the composite efficacy outcomes (Fig. 1a, b).

**Fig. 2:**
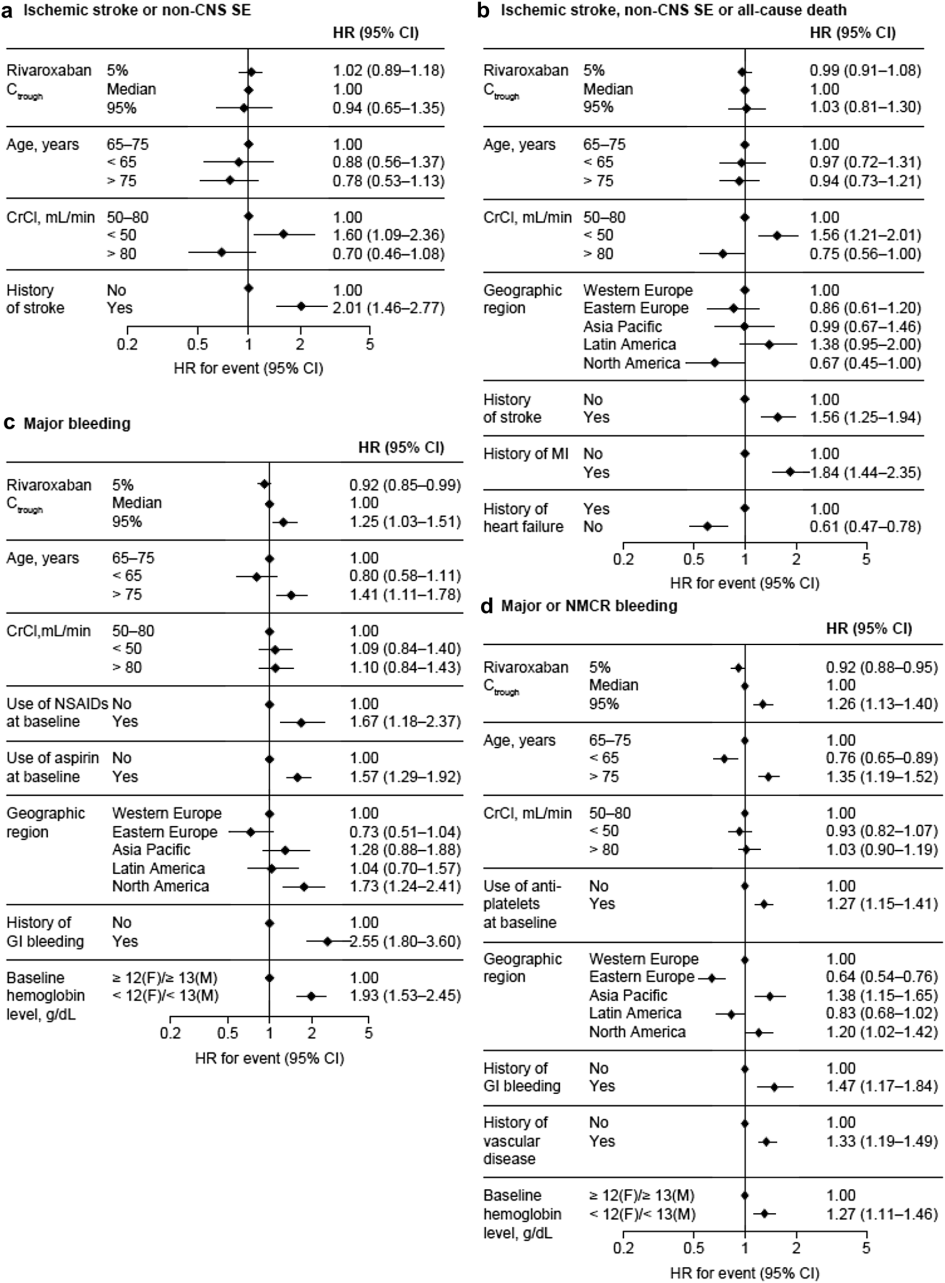
HRs for the composite efficacy outcomes of (**a**) ischemic stroke or non-CNS SE and (**b**) ischemic stroke, non-CNS SE or all-cause death, based on results of the final model; and HRs for the safety outcomes of (**c**) major bleeding and (**d**) major or NMCR bleeding, based on results of the final model. *CI* confidence interval, *CNS* central nervous system, *CrCl* creatinine clearance, *C*_*trough*_ trough plasma concentration, *F* female, *GI* gastrointestinal, *HR* hazard ratio, *M* male, *MI* myocardial infarction, *NMCR* non-major clinically relevant, *NSAID* non-steroidal anti-inflammatory drug, *SE* systemic embolism

There was also no significant association between C_trough_ and the outcome in the final model for ischemic stroke or non-CNS SE; the HRs associated with C_trough_ in the 5th and 95th percentiles versus the median were 1.02 (95% confidence interval [CI] 0.89–1.18) and 0.94 (95% CI 0.65–1.35), respectively (Fig. 2a). Of the variables included in the model, CrCl and history of stroke showed a significant association with the outcome; there was no significant association with age (Fig. 2a, Supplemental Table 3).

In the final model for ischemic stroke, non-CNS SE or all-cause death, there were no significant associations between either C_trough_ or age and the outcome; significant associations were evident for CrCl, geographic region and histories of stroke, myocardial infarction (MI) and heart failure (Fig. 2b, Supplemental Table 3). Histories of stroke and MI had an impact similar to or greater than CrCl, with HRs of 1.56 (95% CI 1.25–1.94) and 1.84 (95% CI 1.44–2.35), respectively.

**Fig. 3:**
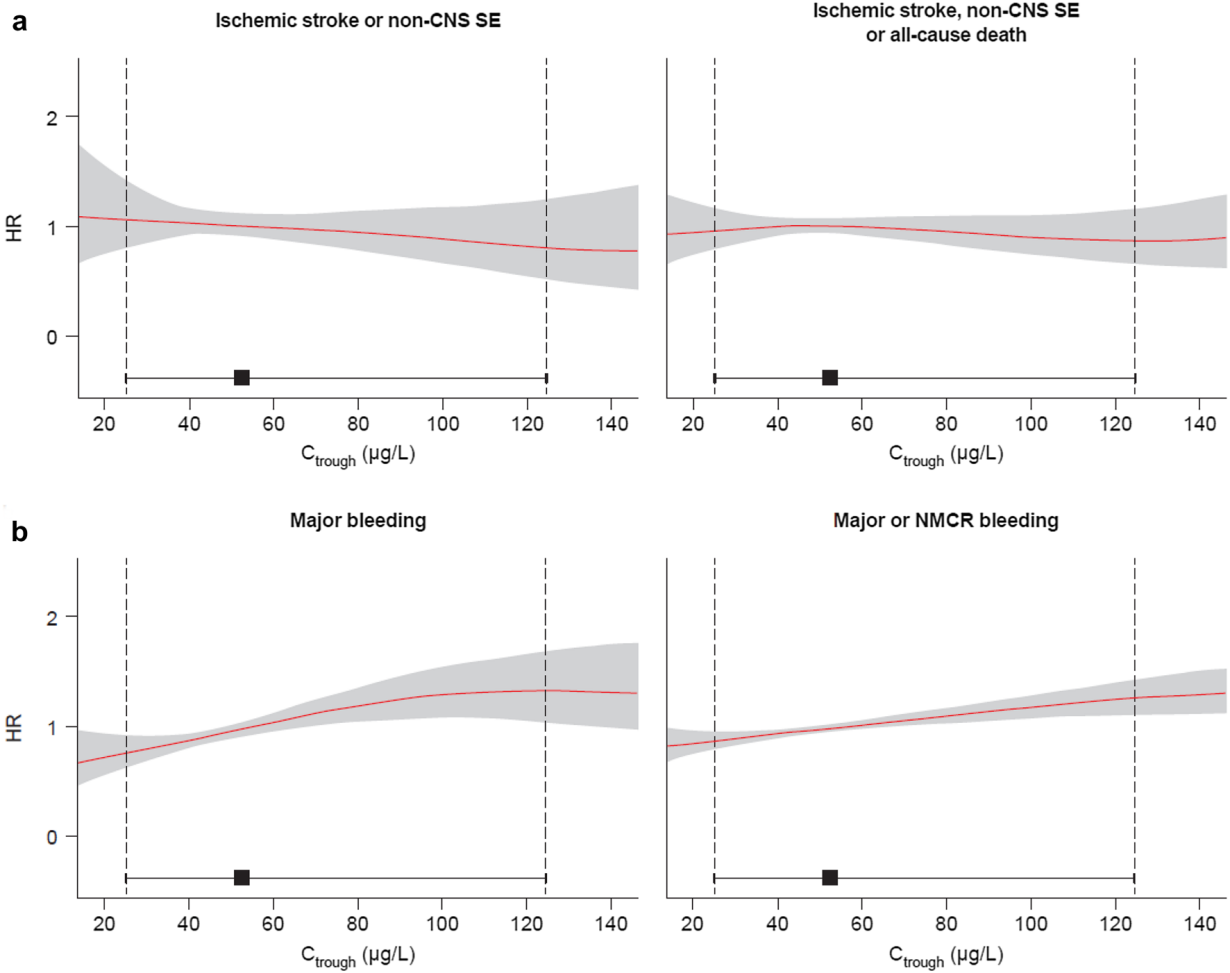
Expected HRs for (**a**) efficacy and (**b**) safety outcomes in a typical patient plotted against the range of predicted C_trough_ values. Red lines represent means and shaded areas represent 95% confidence intervals. Black squares represent median C_trough_ and horizontal error bars represent the range between the 5th and 95th percentiles of C_trough_. Vertical dashed lines label the 5th and 95th percentiles of C_trough_. *CNS* central nervous system, *C*_*troug*h_ trough plasma concentration, *HR* hazard ratio, *NMCR* non-major clinically relevant, *SE* systemic embolism

There was a small decrease in expected HR for ischemic stroke or non-CNS SE with increasing predicted C_trough_ values (Fig. 3a). The association was relatively flat between the HR for ischemic stroke, non-CNS SE or all-cause death and predicted C_trough_ values (Fig. 3a).

#### Exposure–safety analysis

The cumulative event rates for major bleeding (Fig. 1c) and for the composite of major or NMCR bleeding (Fig. 1d) increased with increasing rivaroxaban C_trough_.

In the final model for major bleeding, the HRs associated with C_trough_ in the 5th and 95th percentiles (vs. the median) were 0.92 (95% CI 0.85–0.99) and 1.25 (95% CI 1.03–1.51), respectively; the association between C_trough_ and major bleeding risk was not statistically significant (Fig. 2c). Age (> 75 years vs. 65–75 years) was significantly associated with major bleeding (Fig. 2c, Supplemental Table 3). Patients in North America versus Western Europe had a higher risk of major bleeding, and the risk of major bleeding was higher in patients with versus without baseline use of NSAIDs or aspirin, a history of gastrointestinal (GI) bleeding, and low baseline hemoglobin. CrCl had no significant impact on major bleeding risk.

For major or NMCR bleeding, the HRs associated with C_trough_ in the 5th and 95th percentiles (vs. the median) in the final model were statistically significant (0.92 [95% CI 0.88–0.95] and 1.26 [95% CI 1.13–1.40], respectively; Fig. 2d, Supplemental Table 3). Overall, history of GI bleeding had the greatest impact on this outcome. Patients aged > 75 years were more likely to experience major or NMCR bleeding than those aged 65–75 years, as were patients with versus without low baseline hemoglobin, antiplatelet therapy or a history of vascular disease. The magnitude of the impact of these covariates on the risk of major or NMCR bleeding was similar or greater than that of rivaroxaban C_trough_.

For major bleeding, there was a small increase in HR with increasing C_trough_ values, which appeared to plateau at ~ 115 µg/L (Fig. 3b). For major or NMCR bleeding, there was a small increase in HR over the range of C_trough_ values (Fig. 3b).

#### Expected probability of efficacy or safety events at 1 year of treatment with rivaroxaban

An increase in C_trough_ from the median to the 95th percentile was predicted to increase the probability of having a major bleeding event from ~ 2.1 to ~ 2.8% (p = 0.0211) and the probability of having a major or NMCR bleeding event from ~ 12.2 to ~ 15.5% (p = 0.00002) (Supplemental Fig. 1). Having a history of GI bleeding shifted the entire exposure–response curve for major bleeding upwards and appeared to have a greater impact on the probability of major bleeding at 1 year of treatment than any of the predicted changes in rivaroxaban exposure. An increase in C_trough_ from the median to the 95th percentile was predicted to increase the probability of having a major bleeding event from ~ 2.1 to ~ 2.8% in patients without a history of GI bleeding, and from ~ 5.1 to ~ 6.9% in patients with a history of GI bleeding.

## Discussion

This analysis evaluated rivaroxaban exposure–response relationships in over 7000 patients with NVAF to assess the potential of monitoring drug levels and evaluating patient characteristics in optimizing the benefit–risk profile of treatment.

Warfarin, which requires monitoring, has a clear delineation between international normalized ratio values that are associated with maximum efficacy and those that are associated with increased bleeding risk (i.e. a narrow therapeutic window) [16].

In this analysis, rivaroxaban showed no clear lower limit of exposure that resulted in loss of efficacy, indicating a wide therapeutic window for efficacy in the NVAF indication. Several patient characteristics were significantly associated with the composite efficacy outcomes, but the CHADS_2_ score showed no significant association. A likely explanation is that history of stroke (which showed significant associations with both composite efficacy outcomes) was included as an independent risk factor in the model. Impaired renal function (CrCl < 50 mL/min) showed significant associations with both composite efficacy outcomes.

Increasing predicted rivaroxaban C_trough_ from the median to the 95th percentile was associated with a significant increase in the risk of major or NMCR bleeding, with a HR of 1.26. The HR for major bleeding was similar (1.25) but the association between C_trough_ and the risk of major bleeding was not statistically significant. This may reflect the smaller number of major bleeding events compared with the composite of major or NMCR bleeding events (395 vs. 1475). Thus, the significance of the association between rivaroxaban exposure and major bleeding and the extent to which this contributes to the association between exposure and the composite of major or NMCR bleeding remains uncertain. However, the present analysis does show that the exposure–response relationships for both major bleeding and the composite of major or NMCR bleeding were shallow, with a gradual increase in bleeding risk across a wide range of predicted exposures and no clear threshold of exposure above which the increase in bleeding risk accelerated. The expected increase in the HR of the composite of major or NMCR bleeding, and possibly major bleeding, is therefore small relative to the change in rivaroxaban plasma concentration, which means that any potential gain from measuring rivaroxaban levels and forcing a change in dose would be limited. The CIs around the 1-year estimates of bleeding event rates were wide for any given rivaroxaban concentration and overlapped within the 5th and 95th percentiles of exposure. Taken together, these results suggest that therapeutic drug monitoring would be of limited benefit in patients with NVAF receiving rivaroxaban under the prescribed regimen.

Our analysis identified age, NSAID or aspirin use, history of GI bleeding and low baseline hemoglobin as the components of the HAS-BLED and other bleeding scores [4, 8, 11], which were statistically significant risk factors for major bleeding. These patient characteristics therefore appeared to be more important determinants of risk than rivaroxaban exposure. The increased risk of major bleeding in North American patients compared with those from Western Europe observed in this analysis may be due to ascertainment bias or other confounding factors, such as comorbidities [12]. For major or NMCR bleeding, patient characteristics such as history of GI bleeding and age were statistically significant risk factors, with an impact similar to or greater than rivaroxaban exposure. For example, increasing rivaroxaban C_trough_ from the median to the 95th percentile (from 52.55 to 124.13 µg/L) increased the risk of the composite of major or NMCR bleeding by 26%, whereas having a history of GI bleeding increased this risk by 47%.

Similar findings regarding the effects of exposure and patient characteristics on bleeding risk have been reported for edoxaban, another direct factor Xa inhibitor. In separate analyses of phase 2 and phase 3 trial data, there were significant increases in bleeding risk with increasing edoxaban exposure in patients with NVAF [17, 18]. In contrast to the present results for rivaroxaban, the relationship between edoxaban exposure and bleeding risk was steep over the exposure range [18]. However, edoxaban dose reductions based on patient characteristics in the phase three trial were associated with preservation of efficacy and further reductions in the incidence of major bleeding compared with warfarin (dose reduction vs. no dose reduction; p interaction ≤ 0.023), leading the authors to conclude that the data validate the strategy of tailoring the dose based on clinical factors alone and that such a strategy obviates the need for drug monitoring [19]. The significant variability in exposures in both the edoxaban and dabigatran trials and thus the potential difficulty in selecting threshold drug concentrations for guiding dose changes was also highlighted [18, 19].

The dosage of rivaroxaban in ROCKET AF was tailored based on renal function (20 mg OD reduced to 15 mg OD in patients with a CrCl of 30–49 mL/min) and these dosages were subsequently approved for the NVAF indication [2, 20]. Renal function is also a key consideration in decision-making regarding peri-procedural management of rivaroxaban therapy [21]. While monitoring of coagulation and plasma drug concentrations has been proposed in some patients for guiding pre- and peri-procedural management of direct oral anticoagulants [22], expert consensus from the American College of Cardiology (ACC) focuses on the importance of patient and procedural risk factors. The ACC recommends that patient risk factors for bleeding followed by bleeding risk of the procedure be considered for the decision on whether or not to interrupt therapy, and that the specific drug and level of renal function then be used to guide the timing and duration of interruption to therapy [21]. Results from the present analysis support the central role of patient characteristics in decision-making processes regarding bleeding risk with rivaroxaban and the limited likely value of adding drug monitoring into management pathways.

Limitations of this analysis include the paucity of direct rivaroxaban plasma concentration measurements in ROCKET AF, although this was partially offset by the PT adjustment in some patients [14, 15]. The predicted C_trough_ values showed moderate between-patient variability (coefficient of variation: 54%) and were consistent with the previously published ROCKET AF popPK model [23]. In addition, because ROCKET AF was not designed to evaluate exposure–response relationships, the current analysis may have been underpowered to detect statistically significant differences for some outcomes. Finally, the exposure–response analysis included baseline use of antiplatelet agents and NSAIDs but did not evaluate the impact of their continued use during follow-up.

## Conclusions

These results support fixed rivaroxaban 15 mg and 20 mg OD dosages in patients with NVAF and suggest therapeutic drug monitoring is unlikely to offer clinical benefits in this indication beyond evaluation of patient characteristics.

## Electronic supplementary material

Below is the link to the electronic supplementary material.

Supplementary file1 (DOCX 107 kb)
