# Supplementary material for: Associations between model-predicted rivaroxaban exposure and patient characteristics and efficacy and safety outcomes in patients with non-valvular atrial fibrillation
Source: J Thromb Thrombolysis. 2020 Apr 23;50(1):20–9. doi: 10.1007/s11239-020-02077-9 (PMC7293978; doi:10.1007/s11239-020-02077-9)
Supplement: Supplementary file 1 — Supplementary file1 (DOCX 107 kb) [file 11239_2020_2077_MOESM1_ESM.docx]

Supplemental Appendix

**Associations between model-predicted rivaroxaban exposure and patient characteristics and efficacy and safety outcomes in patients with non-valvular atrial fibrillation**

*Journal of Thrombosis and Thrombolysis*

Liping Zhang^1^ • Xiaoyu Yan^1^ • Keith A. A. Fox^2^ • Stefan Willmann^3^ • Partha Nandy^1^ • Scott D. Berkowitz^4^ • Anne Hermanowski-Vosatka^1^ • Jeffrey I. Weitz^5^ • Alexander Solms^6^ • Stephan Schmidt^7^ • Manesh Patel^8^ • Gary Peters^1^

^1^ Janssen Research & Development, LLC, Raritan, NJ, USA

^2^ Centre for Cardiovascular Science, The University of Edinburgh, Edinburgh, UK

^3^ Clinical Pharmacometrics, Bayer AG, Wuppertal, Germany

^4^ Bayer U.S., LLC, Research & Development, Pharmaceuticals, Whippany, NJ, USA

^5^ Department of Medicine, McMaster University, and the Thrombosis & Atherosclerosis Research Institute, Hamilton, ON, Canada

^6^ Clinical Pharmacometrics, Bayer AG, Berlin, Germany

^7^ Center for Pharmacometrics and Systems Pharmacology, Department of Pharmaceutics, College of Pharmacy, University of Florida, Orlando, FL, USA

^8^ Duke Clinical Research Institute, Durham, NC, USA

**Address for correspondence** Liping Zhang, Senior Scientific Director, Clinical Pharmacology and Pharmacometrics, Janssen Research & Development, LLC, 920 Route 202, Raritan, NJ 08869, USA

Tel.: +1 609 619 2430

E-mail: [LZhang11@ITS.JNJ.com](mailto:LZhang11@ITS.JNJ.com)

**Methods**

**Regression analyses**

Relationships between rivaroxaban exposure metrics, patient characteristics and the efficacy and safety outcomes were quantified first by performing univariate Cox proportional hazard regression analysis using area under the plasma concentration–time curve from time 0 to 24 hours (AUC_0–24_), maximum plasma concentration (C_max_) and trough plasma concentration (C_trough_) as independent variables, assuming a linear relationship with the log-hazard of outcome events. The exposure metric showing the strongest association with the likelihood of an event (lowest Akaike information criterion [AIC] value [1]) was then combined with the selected patient characteristics, as independent variables for predicting the probability of the outcome events, in a multivariate Cox proportional regression analysis. The statistical significance of each independent variable was determined by fitting the full model. Statistically non-significant variables (p > 0.01, according to the likelihood ratio test) were removed from the model, with the exception of the selected exposure metric, age and creatinine clearance (calculated using the Cockcroft–Gault formula); these were expected to influence the outcome events and were therefore retained as forced input variables, regardless of their associated p values. The derived model, containing the forced input variables and statistically significant variables, was considered the final model for the evaluated response outcome.

**Results**

**Selection of exposure metric for investigation in exposure–response models**

In the univariate regression analysis, the AIC values estimated from fitting the exposure metrics (AUC_0–24_, C_max_ and C_trough_) differed only marginally owing to the high correlation between predicted AUC_0–24_, C_max_ and C_trough_. However, C_trough_ was associated with the lowest AIC value and was selected for further investigation in the exposure–efficacy and exposure–safety models.

**Reference**

1. Olofsen E, Dahan A (2013) Using Akaike's information theoretic criterion in mixed-effects modeling of pharmacokinetic data: a simulation study. F1000Res 2:71. doi:10.12688/f1000research.2-71.v2

## **Supplemental Table 1** Definition and counts of patient characteristics for inclusion in exposure–response models

| **Covariate** | **Categories^a^** | **Exposure–efficacy models** | | **Exposure–safety models** | |
| --- | --- | --- | --- | --- | --- |
|  |  | **Number of patients (n = 7061)** | **Percent^b^** | **Number of patients (n = 7111)** | **Percent^b^** |
| Patient characteristics included in the exposure–response models regardless of significance level | | | | | |
| Age | **65–75 years** | **2767** | **39.2** | **2777** | **39.1** |
|  | > 75 years | 2652 | 37.6 | 2688 | 37.8 |
|  | < 65 years | 1642 | 23.3 | 1646 | 23.1 |
| Baseline renal function (CrCl) | **50–80 mL/min** | **3297** | **46.7** | **3320** | **46.7** |
|  | > 80 mL/min | 2279 | 32.3 | 2289 | 32.2 |
|  | < 50 mL/min | 1485 | 21.0 | 1502 | 21.1 |
| Patient characteristics for potential inclusion in exposure–response models | | | | | |
| Baseline antiplatelet use  (including aspirin, thienopyridine, aspirin/dipyridamole, etc.^c^) | **No** | **4226** | **59.8** | **4263** | **59.9** |
|  | Yes | 2835 | 40.2 | 2848 | 40.1 |
| Baseline NSAID use | **No** | **6780** | **96.0** | **6830** | **96.0** |
|  | Yes | 281 | 4.0 | 281 | 4.0 |
| Baseline chronic aspirin use | **No** | **4494** | **63.6** | **4533** | **63.7** |
|  | Yes | 2567 | 36.4 | 2578 | 36.3 |
| Prior VKA use | **Yes** | **4401** | **62.3** | **4431** | **62.3** |
|  | No | 2660 | 37.7 | 2680 | 37.7 |
| History of stroke | **No** | **4632** | **65.6** | **4670** | **65.7** |
|  | Yes | 2429 | 34.4 | 2441 | 34.3 |
| History of TIA | **No** | **5501** | **77.9** | **5541** | **77.9** |
|  | Yes | 1560 | 22.1 | 1570 | 22.1 |
| History of systemic embolism | **No** | **6788** | **96.1** | **6835** | **96.1** |
|  | Yes | 273 | 3.9 | 276 | 3.9 |
| Geographic region | Eastern Europe | 2696 | 38.2 | 2746 | 38.6 |
|  | North America | 1334 | 18.9 | 1334 | 18.8 |
|  | Asia Pacific | 1052 | 14.9 | 1052 | 14.8 |
|  | **Western Europe** | **1040** | **14.7** | **1040** | **14.6** |
|  | Latin America | 939 | 13.3 | 939 | 13.2 |
| Weight | **≥ 60 and < 90 kg** | **4201** | **59.5** | **4238** | **59.6** |
|  | ≥ 90 kg | 2185 | 30.9 | 2197 | 30.9 |
|  | < 60 kg | 675 | 9.6 | 676 | 9.5 |
| Sex | **Male** | **4270** | **60.5** | **4292** | **60.4** |
|  | Female | 2791 | 39.5 | 2819 | 39.6 |
| Baseline diastolic blood pressure | **< 90 mmHg** | **5647** | **80.0** | **5680** | **79.9** |
|  | ≥ 90 mmHg | 1414 | 20.0 | 1431 | 20.1 |
| History of COPD | **No** | **6317** | **89.5** | **6360** | **89.4** |
|  | Yes | 744 | 10.5 | 751 | 10.6 |
| History of GI bleeding | **No** | **6836** | **96.8** | **6883** | **96.8** |
|  | Yes | 225 | 3.2 | 228 | 3.2 |
| History of ICH | **No** | **7054** | **99.9** | **7104** | **99.9** |
|  | Yes | 7 | 0.1 | 7 | 0.1 |
| Atrial fibrillation | **Persistent** | **5739** | **81.3** | **5771** | **81.2** |
|  | Paroxysmal | 1228 | 17.4 | 1242 | 17.5 |
|  | New | 94 | 1.3 | 98 | 1.4 |
| History of MI | **No** | **5892** | **83.4** | **5933** | **83.4** |
|  | Yes | 1169 | 16.6 | 1178 | 16.6 |
| History of vascular disease  (CAD, PAD or COD) | **No** | **5133** | **72.7** | **5164** | **72.6** |
|  | Yes | 1928 | 27.3 | 1947 | 27.4 |
| Heart rate from baseline ECG | **≥ 60 and < 100 BPM** | **5400** | **76.5** | **5436** | **76.4** |
|  | ≥ 100 BPM | 957 | 13.6 | 969 | 13.6 |
|  | < 60 BPM | 704 | 10.0 | 706 | 9.9 |
| History of diabetes mellitus | **No** | **4219** | **59.8** | **4242** | **59.7** |
|  | Yes | 2842 | 40.2 | 2869 | 40.3 |
| History of heart failure | **Yes** | **4485** | **63.5** | **4515** | **63.5** |
|  | No | 2576 | 36.5 | 2596 | 36.5 |
| History of hypertension | **Yes** | **6372** | **90.2** | **6419** | **90.3** |
|  | No | 689 | 9.8 | 692 | 9.7 |
| Alcohol use | **Abstinent** | **4548** | **64.4** | **4576** | **64.4** |
|  | Light | 2152 | 30.5 | 2173 | 30.6 |
|  | Moderate | 306 | 4.3 | 307 | 4.3 |
|  | Heavy | 55 | 0.8 | 55 | 0.8 |
| Smoking status | **No** | **6659** | **94.3** | **6706** | **94.3** |
|  | Yes | 402 | 5.7 | 405 | 5.7 |
| Low baseline hemoglobin  (< 13 g/dL for men;  < 12 g/dL for women) | **No** | **6090** | **86.2** | **6132** | **86.2** |
|  | Yes | 971 | 13.8 | 979 | 13.8 |
| CHADS_2_ score | **3** | **3025** | **42.8** | **3047** | **42.8** |
|  | 4 | 2073 | 29.4 | 2087 | 29.3 |
|  | 2 | 922 | 13.1 | 923 | 13.0 |
|  | 5 | 918 | 13.0 | 930 | 13.1 |
|  | 6 | 122 | 1.7 | 123 | 1.7 |
|  | 1 | 1 | < 0.1 | 1 | < 0.1 |

*BPM* beats per minute, *CAD* coronary artery disease, *CHADS_2_* congestive heart failure, hypertension, age ≥ 75 years, diabetes mellitus, stroke (double weight), *COD* carotid occlusive disease, *COPD* chronic obstructive pulmonary disease, *CrCl* creatinine clearance, *ECG* electrocardiogram, *GI* gastrointestinal, *ICH* intracranial hemorrhage, *MI* myocardial infarction, *NSAID* non-steroidal anti‑inflammatory drug, *PAD* peripheral artery disease, *TIA* transient ischemic attack, *VKA* vitamin K antagonist

^a^For each covariate, the reference category is highlighted in bold font

^b^Percentages are rounded to the nearest 0.1%

^c^Baseline antiplatelet medications were abciximab, acetylsalicylate lysine, acetylsalicylic acid (including acetylsalicylic acid with glycerol, magnesium oxide or magnesium hydroxide), acidum acetylsalicylicum, albyl-enterosoluble, anagrelide hydrochloride, acetylsalicylic acid or aspirin with dipyridamole, aspirin with magnesium oxide, aspirin, beraprost sodium, carbasalate calcium, cilostazol, clopidogrel (including clopidogrel sulfate or bisulfate), dipyridamole, eicosapentaenoic acid, epoprostenol, eptifibatide, iloprost, iloprost trometamol, indobufen, mesoglycan, ozagrel sodium, platelet aggregation inhibitors, sarpogrelate, sarpogrelate hydrochloride, ticlopidine, ticlopidine hydrochloride, tirofiban hydrochloride or triflusal

## **Supplemental Table 2** Summary of predicted rivaroxaban exposure in the efficacy and safety populations of the ROCKET AF study

| **Population** | **Exposure measure** | **n** | **P05** | **Median** | **P95** | **Mean** | **CV (%)** |
| --- | --- | --- | --- | --- | --- | --- | --- |
| Efficacy population | AUC_0–24_ (µg/L×h) | 7061 | 2445.30 | 3377.20 | 5328.20 | 3554.06 | 26.01 |
|  | C_max_ (µg/L) | 7061 | 205.70 | 255.40 | 347.40 | 262.63 | 17.14 |
|  | C_trough_ (µg/L) | 7061 | 25.26 | 52.51 | 124.14 | 60.32 | 54.11 |
| Safety population | AUC_0–24_ (µg/L×h) | 7111 | 2448.75 | 3379.40 | 5328.95 | 3555.45 | 25.99 |
|  | C_max_ (µg/L) | 7111 | 205.70 | 255.50 | 347.60 | 262.69 | 17.13 |
|  | C_trough_ (µg/L) | 7111 | 25.27 | 52.55 | 124.13 | 60.36 | 54.04 |

*AUC_0–24_* area under the concentration–time curve from 0 to 24 hours, *C_max_* maximum plasma concentration, *C_trough_* trough plasma concentration, *CV* coefficient of variation,
*P05* 5th percentile, *P95* 95th percentile

**Supplemental Table 3** Results of the likelihood ratio test for the final models

| **Variable** | **Degree of freedom** | **AIC** | **p value (likelihood ratio test)** |
| --- | --- | --- | --- |
| **Ischemic stroke or non-CNS SE** | | | |
| Age | 2 | 2584.175 | 0.47197 |
| CrCl | 2 | 2593.361 | 0.00478 |
| Stroke | 1 | 2605.695 | < 0.0001 |
| **Ischemic stroke, non-CNS SE or all-cause death** | | | |
| Age | 2 | 5976.166 | 0.87636 |
| CrCl | 2 | 5995.540 | 0.00005 |
| Geographic region | 4 | 5987.587 | 0.00347 |
| Stroke | 1 | 5987.344 | < 0.0001 |
| History of MI | 1 | 5999.435 | < 0.00001 |
| History of heart failure | 1 | 5993.883 | 0.00006 |
| **Major bleeding** | | | |
| Age | 2 | 6505.709 | 0.0012 |
| CrCl | 2 | 6492.577 | 0.85049 |
| NSAID use at baseline | 1 | 6501.146 | 0.00865 |
| Aspirin use at baseline | 1 | 6514.128 | 0.00001 |
| Geographic region | 4 | 6533.364 | < 0.00001 |
| GI bleeding | 1 | 6516.957 | < 0.00001 |
| Baseline hemoglobin | 1 | 6525.122 | < 0.00001 |
| **Major or NMCR bleeding** | | | |
| C_trough_ | 1 | 24,633.74 | 0.00003 |
| Age | 2 | 24,661.05 | < 0.00001 |
| CrCl | 2 | 24,615.84 | 0.46286 |
| Antiplatelet use at baseline | 1 | 24,636.13 | 0.00001 |
| Geographic region | 4 | 24,733.12 | < 0.00001 |
| GI bleeding | 1 | 24,626.22 | 0.00163 |
| History of vascular disease | 1 | 24,639.66 | < 0.00001 |
| Baseline hemoglobin | 1 | 24,627.54 | 0.0008 |
| *AIC* Akaike information criterion, *CNS* central nervous system, *CrCl* creatinine clearance, *C_trough_* trough concentration, *GI* gastrointestinal, *MI* myocardial infarction, *NMCR* non-major clinically relevant, *NSAID* non-steroidal anti-inflammatory drug, *SE* systemic embolism | | | |

**
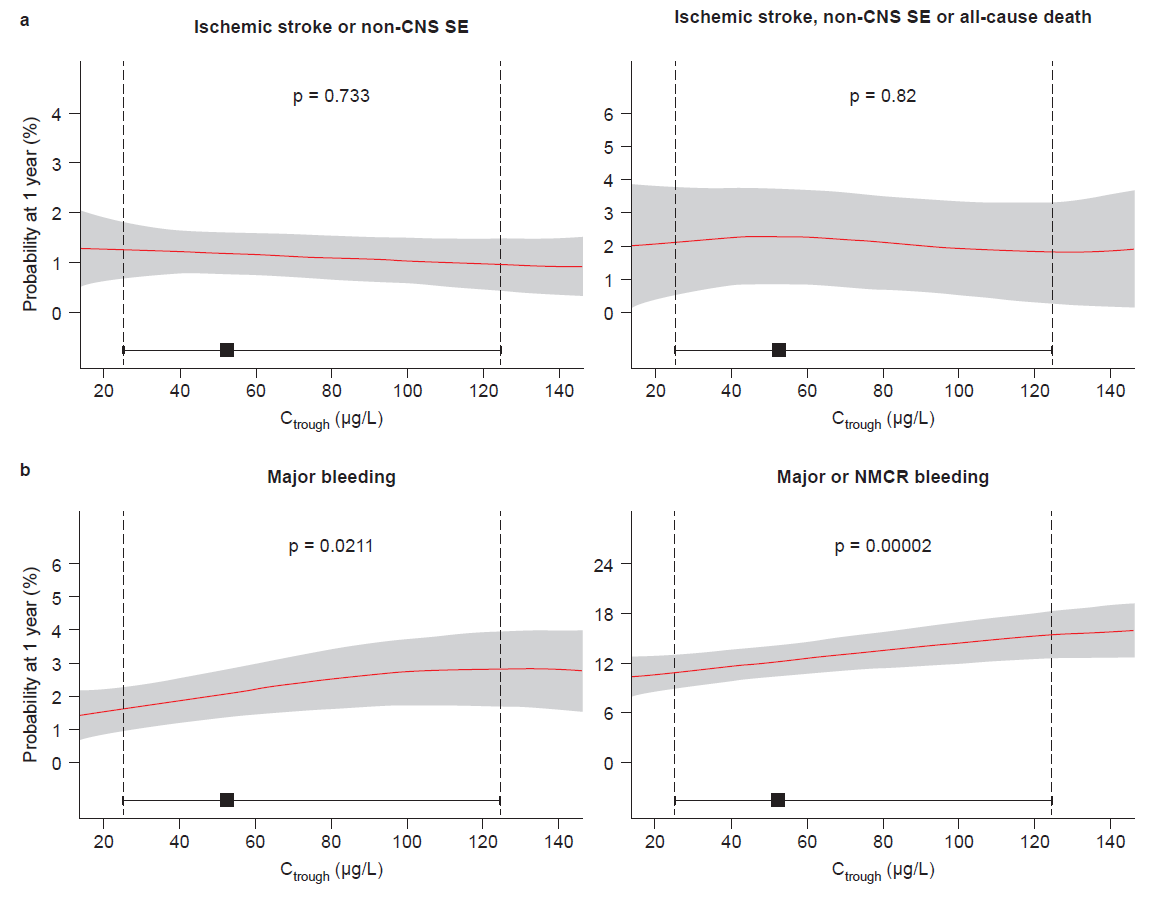
Supplemental Fig. 1** Expected probability of (**a**) efficacy and (**b**) safety events at 1 year of treatment with rivaroxaban in a typical patient plotted against the range of increasing C_trough_ values. Red lines represent means and shaded areas represent 95% confidence intervals. Black squares represent median C_trough_ and horizontal error bars represent the range between the 5th and 95th percentiles of C_trough_. Vertical dashed lines label the 5th and 95th percentiles of C_trough_. p values presented are for the comparison of the 95th percentile of C_trough_ with the median. *CNS* central nervous system, *C_trough_* trough concentration, *NMCR* non-major clinically relevant, *SE* systemic embolism
